# Therapeutic Applications of Carbon Monoxide

**DOI:** 10.1155/2013/360815

**Published:** 2013-12-04

**Authors:** Melissa Knauert, Sandeep Vangala, Maria Haslip, Patty J. Lee

**Affiliations:** Section of Pulmonary, Critical Care and Sleep Medicine, Yale University School of Medicine, 300 Cedar Street, TAC-441 South, P.O. Box 208057, New Haven, CT 06520-8057, USA

## Abstract

Heme oxygenase-1 (HO-1) is a regulated enzyme induced in multiple stress states. Carbon monoxide (CO) is a product of HO catalysis of heme. In many circumstances, CO appears to functionally replace HO-1, and CO is known to have endogenous anti-inflammatory, anti-apoptotic, and antiproliferative effects. CO is well studied in anoxia-reoxygenation and ischemia-reperfusion models and has advanced to phase II trials for treatment of several clinical entities. In alternative injury models, laboratories have used sepsis, acute lung injury, and systemic inflammatory challenges to assess the ability of CO to rescue cells, organs, and organisms. Hopefully, the research supporting the protective effects of CO in animal models will translate into therapeutic benefits for patients. Preclinical studies of CO are now moving towards more complex damage models that reflect polymicrobial sepsis or two-step injuries, such as sepsis complicated by acute respiratory distress syndrome. Furthermore, co-treatment and post-treatment with CO are being explored in which the insult occurs before there is an opportunity to intervene therapeutically. The aim of this review is to discuss the potential therapeutic implications of CO with a focus on lung injury and sepsis-related models.

## 1. Introduction

Inducible heme oxygenase, heme oxygenase-1 (HO-1), is a regulated enzyme that is induced in response to oxidative stress. HO-1 catalysis is the rate limiting step in the breakdown of heme, a powerful intracellular catalyst of free radical production, to equimolar amounts of carbon monoxide (CO), biliverdin, and iron. Biliverdin is immediately converted to bilirubin and iron is rapidly sequestered into ferritin. CO remains behind as a stable, diffusible molecule with potent cell signaling capabilities. Experimentally we know that diverse insults such as ischemia, hypoxia, hyperoxia, endotoxin exposure, polymicrobial infection, ventilator induced lung injury, hemorrhage, and transplant cause HO-1 upregulation at the transcriptional and, in a few cases, translational level.

In parallel with the elevation in HO-1, CO levels are increased in multiple disease states such as asthma [1], cystic fibrosis [2], and sepsis [3]. Multiple experimental models have demonstrated that the pleiotropic effects of HO-1 can be mimicked via the application of exogenous CO. Given its safety in low doses [4–6] and relative ease of administration [7] compared to therapeutic HO-1 gene strategies, CO has been proposed as a therapeutic entity. This technology has already advanced as far as phase II trials for postoperative patients and patients with idiopathic pulmonary fibrosis, pulmonary hypertension, and chronic obstructive pulmonary disease (COPD). Because there is accumulating data regarding the ability of CO to abrogate primary and secondary acute lung injury (ALI) and control systemic inflammatory damage attributed to sepsis, there may one day be uses in the critical care setting.

CO, the diatomic oxide of carbon, is a colorless, ubiquitous gas at temperatures above −190°C. It has a specific gravity of 0.967 relative to air and a density of 1.25 g/L at standard temperature and pressure [8]. CO readily forms metal carbonyls, which are susceptible to the attack of the CO oxygen atom by electrophiles. Chemical reduction of CO, however, requires temperatures well above the normal physiological range (>100°C); metal carbonyls are relatively stable until CO is displaced, for example, by molecular oxygen [9]. Most of the CO in the body (80%) is bound to hemoglobin as COHb [10]. The remainder of the CO is distributed in the tissues. The cellular concentrations of CO depend upon the local partial pressures of both CO and oxygen because the two gases compete for the same iron or copper binding sites. Among cellular heme proteins, myoglobin, cytochrome c oxidase, cytochrome p450, catalase, guanylate cyclase, and tryptophan dioxygenase bind sufficient CO to alter function *in vitro*. After binding to heme protein enzymes, CO usually inhibits electron transfer and/or catalytic activity [11].

Multiple actions of CO, of both exogenous and endogenous derivations, depend significantly on the concentrations of both CO and reduced transition metals, for example, Fe (II), in relation to the availability of molecular O_2_. In this respect, the CO/O_2_ ratio and O_2_-dependent changes in the redox state of the cell, or in different compartments within a cell, assume critical importance in the effects of CO on specific protein functions. Because CO may influence the reactions involving heme proteins, it can be expected to have both pro-oxidant and antioxidant effects on the cell [12].

**Figure 1:**
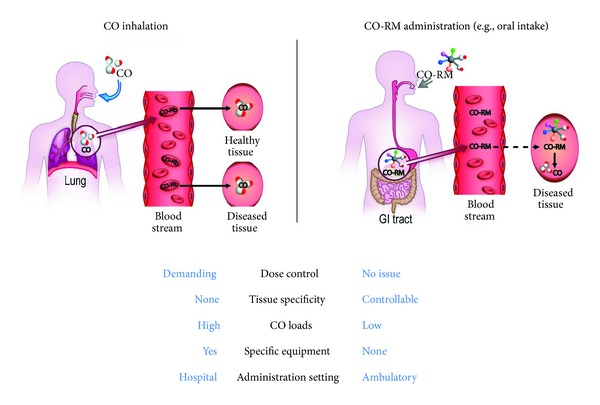
Alternative pathways for the therapeutic delivery of CO to diseased tissues with their main advantages and disadvantages. From Romao et al. (2012) [14].

The only means of ridding the body of CO is through exhalation. CO is best known for its high affinity for hemoglobin and the resultant displacement of oxygen at high concentrations; this causes tissue hypoxia and the associated pathologies of CO poisoning. Maximal tolerated levels are generally considered to be 10–12% and are equivalent to the CO blood levels of heavy smokers. CO dosage is generally discussed in parts per million (ppm) and typical experimental doses of 250 to 500 ppm result in CO serum levels well below 10% [4–6]. As the work to elucidate the physiologic and therapeutic role of gaseous CO has progressed, a parallel line of inquiry has produced a family of molecules known as CO releasing molecules (CO-RMs) capable of carrying and delivering CO to tissues in physiologic conditions [13]. The prototypic members of this family contain a transition metal core which binds (and then releases) CO. Technology has advanced these agents from lipid to water soluble and produced a large variety of compounds with varying potency and half-lives. See Figure 1, Romao et al. [14].

Sepsis is the leading cause of hospital mortality in adult patients and the incidence is increasing [15]. One recent study in the United States revealed 3.0 cases of severe sepsis (defined as sepsis plus organ dysfunction) per 1,000 people and 2.3 cases per 100 hospital discharges; mortality was 28.6% in this cohort [16, 17]. When sepsis is combined with ALI or acute respiratory distress syndrome (ARDS) the outcomes are worse. ARDS associated with sepsis has a higher disease severity, poorer recovery, lower successful extubation rate, and higher mortality as compared to nonsepsis ARDS [18]. These vulnerable patients with sepsis and ARDS may potentially benefit from CO therapies, if safe doses as well as delivery systems can be established.

The goal of this review is to discuss potential therapeutic applications of CO in the clinical settings of sepsis and lung injury. Much of the focus will be upon the beneficial role of gaseous CO, though we will briefly touch on related results from CO-RM therapies. The biologic and therapeutic potentials of biliverdin and bilirubin as well as HO-1 related therapy are beyond the scope of this review.

## 2. Mechanisms of Action

Carbon monoxide has been demonstrated to be dependent upon a variety of cell signaling pathways but neither a comprehensive list nor the precise molecular interactions have been fully worked out. It appears that CO exerts its effect through different pathways in different cell lines and under different conditions [19–22]. Most recently, investigators have revealed a role for HO-1 in the regulation of autophagy and mitochondrial homeostasis (reviewed in [23]). In addition, multiple members of the mitogen-activated protein kinase (MAPK) family are implicated in the anti-inflammatory and anti-apoptotic effects of HO-1 and CO; however, depending on the organ or tissue type, these effects can be dependent or independent of the three major MAPK pathways: p38, extracellular signal-regulated kinase (ERK) 1 and ERK2, and Jun Kinase (JNK). For example, an ERK1/2 MAPK dependent pathway has been shown to be responsible for CO-related inhibition of interleukin (IL) 17 in lung injury models; IL-17 plays a key role in neutrophil predominant inflammation of the lung [24]. In the case of a macrophage cell line and the p38 pathway, the effects of exogenous CO administration after lipopolysaccharide (LPS) insult require both mitochondrial produced reactive oxygen species (ROS) and the p38 MAPK pathway [25]. It may be that this mitochondrial burst of ROS is critical in the reported role of CO as a bactericidal agent. Our laboratory has shown that CO utilizes p38*α* MAPK to attenuate oxidant-induced apoptosis in an ischemia-reperfusion lung injury model via modulation of Fas/Fas ligand, B-cell lymphoma-2 (Bcl-2), and caspase-mediated cell death [26, 27]. Activated phosphatidylinositol-3-kinase (PI3 K/Akt) also further induces HO-1 thereby creating a positive feedback loop [28–30]. The p38 dependent induction of HO-1 and/or CO are believed in some cases to target the p38*α* isoform for degradation. This degradation functionally alters the p38*α* to p38*β* ratios and promotes the dominance of the cytoprotective *β* isoform [31]. More generalized activation of p38 MAPK by HO-1 also induces the expression of B-cell lymphoma-extra large protein via the PI3 K/Akt signal transduction pathway which, in turn, inhibits the intrinsic (mitochondrial) apoptotic pathway [31]. Both HO-1 and CO are protective in models of liver disease in which they prevent phosphorylation of the pro-apoptotic JNK MAPK [32].

In addition to MAPK related signaling, other anti-apoptotic effects of HO-1 are mediated through the nuclear factor *κ*-light chain-enhancer of activated B cells (NF-*κ*B) transcription factor pathway. Via an unknown mechanism, HO-1 downmodulates NF-*κ*B activation vis á vis apoptosis without interfering with the expression of downstream cytoprotective genes [21, 31, 33]. In the case of hypoxia, CO on its own can trigger activation of hypoxia inducible factor-*α* which in turn activates anti-inflammatory transforming growth factor *β* [34]. CO also provided protection against endotoxic shock via reciprocal effects on the inducible nitrous oxide synthase pathway in the lung and liver [35]. Finally, in the case of vascular smooth muscle and neuronal cell stress, CO is believed to exert its effects via increases in cyclic guanosine monophosphate signaling [36, 37]. The antiproliferative effects of HO-1 and CO in smooth muscle cells appear to be mediated via caveolin-1, the major structural protein of caveolae which are key components of endocytosis in cells such as lung endothelial cells, type I pneumocytes, (murine) alveolar macrophages and fibroblasts [38]; this pathway is also being explored vis á vis ventilator-induced lung injury [39].

**Figure 2:**
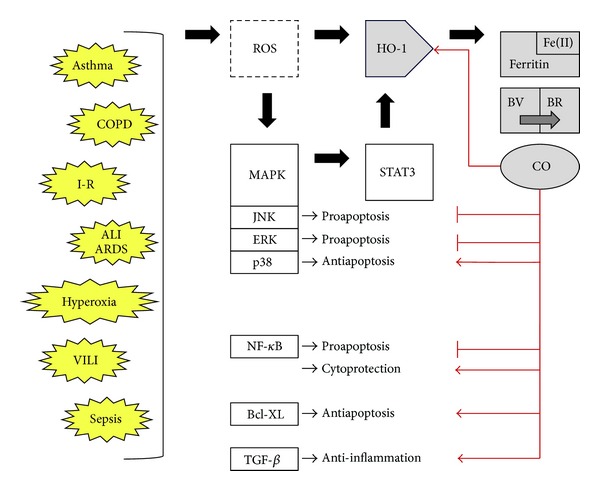
Summary of oxidative stress triggers and HO-1 related cellular responses. BV: biliverdin, BR: bilirubin; otherwise abbreviations are as defined in the text. Oxidative stresses trigger ROS which in turn trigger the MAPK pathway as well as HO-1 via MAPK dependent and independent mechanisms. MAPK further signals through STAT3 to upregulate HO-1 transcription. HO-1 produces free iron which is sequestered into ferritin, biliverdin which is converted into bilirubin, and CO which is stable and diffuses readily. CO production further increases HO-1 levels. In addition, CO influences multiple other signaling pathways to decrease apoptosis and inflammation.

Though it is clear that many of the cell's most central signaling cascades are involved, precise molecular mechanisms are unknown to date and HO-1 signaling interactions appear to be highly situational. Analysis of HO-1 and CO pathways is further complicated by the extensive redundancy of cellular danger and damage signaling. What seems most apparent is the unifying theme of reactive oxygen species as a critical trigger to HO-1 induction and the ability of its product, CO, to abrogate the deleterious effects of an overexuberant inflammatory response via stimulation of cytoprotective and anti-apoptotic pathways. This ambiguous and complex interplay of injury models, cellular stress, signaling molecules, and HO-1 and its product CO are depicted in Figure 2.

## 3. Sepsis Models: Lipopolysaccharide Endotoxemia and Polymicrobial Sepsis

The experimental models discussed below emulate both gram-negative sepsis and associated ALI. Sepsis itself may be an important target for CO therapy. And, in the case of systemically administered LPS, the associated lung injury mimics the ARDS we often see in septic patients as a secondary inflammatory injury. Intranasal and intratracheal LPS administration create direct lung tissue injury, apoptosis, and necrosis, as would be seen in infectious pneumonia [40].

Early investigations into sepsis-related HO-1 effects revealed that HO-1 is induced *in vitro *and *in vivo *in response to a variety of oxidative stresses. Intravenous LPS administration of 0.1 mg/kg in a mouse model resulted in transcriptional upregulation of HO-1 in the kidney, liver, and spleen [41]; similarly, intravenous LPS administration of 4 mg/kg in a rat model revealed induction of HO-1 in the smooth muscle of large and small arteries [42]. HO-1 is also upregulated in the lung tissue of rats following LPS exposure [43]. Importantly, HO-1 induction appears to confer tremendous protection against oxidative stress as demonstrated by Otterbein et al. in work exploring a rat model of sepsis. In this series, rats were pretreated with hemoglobin in order to induce HO-1. Following pretreatment, a lethal dose of LPS was administered. When compared to rats not exposed to hemoglobin, the pretreated rats had 100% survival [43]. Kanagawa et al. demonstrated hepatic growth factor dependent HO-1 induction in an intraperitoneal LPS injection rat model; they further demonstrated diminished lung and kidney injury following LPS insult for HO-1 induced animals [44].

Given that CO is one of three cellular products of HO-1 and a potential downstream mediator of HO-1 effects, the possibility that CO could functionally replace HO-1 has been explored [36]. CO abrogated LPS induction of proinflammatory cytokines tumor necrosis factor *α* (TNF-*α*), IL-1*β*, and macrophage inflammatory protein (MIP)-1*β* and augmented LPS induction of anti-inflammatory IL-10 [36, 45]. Expansion of this work demonstrated the beneficial effects of either HO-1 enzyme induction or exogenous CO in abrogating inflammatory and apoptotic pathways following LPS challenge. Within 24 hours after intratracheal administration of LPS in mice, epithelial cell injury and apoptosis in lung macrophages, neutrophils, and alveolar wall can be detected. CO preconditioning with 250 ppm reduced TNF-*α*, IL-1*β*, IL-6, and the aforementioned injury and apoptosis [36, 46]. Sepsis survival in a murine *Staphylococcus aureus* model was significantly enhanced using inhaled CO, 250 ppm daily for 1 hour, and linked mechanistically to HO-1 induction and mitochondrial HO activity through NF-E2-related factor-2 and Akt kinase [47].

LPS sepsis models are helpful as mimics of the host inflammatory response to infection which is often a driving force behind the pathophysiology of clinical sepsis. However, there is possible harm in blunting an inflammatory response designed to facilitate microbial clearance. In a series of suggestive experiments by Chung et al., utilizing a cecal ligation-puncture (CLP) murine model, it was noted that HO-1 deficient mice had increased gastrointestinal tissue destruction, higher mortality and higher levels of bacteremia. In contrast, mice with HO-1 overexpression targeted to vascular smooth muscle cells had improved mortality, and lower levels of bacteremia. When the authors compared survival in HO-1 overexpressing mice after induction of single species bacteremia, it was noted that HO-1 was protective in the case of *Enterococcus faecalis* bacteremia but not in the case of *Escherichia coli* bacteremia. The survival benefit of HO-1 overexpression was then recapitulated with CO-RM administration before and *after *CLP surgery [48]. Works by Su et al. and Otterbein et al. suggest that at least part of the role of CO in bacterial clearance is related to increased bacterial phagocytosis [48, 49]. Furthermore, when CO is delivered via CO-RM, we also see increased *in vitro *bacterial killing (*Escherichia coli* and *Pseudomonas aeruginosa*) and abrogation of the sepsis insult in murine and rat experiments utilizing LPS and CLP sepsis models [50–54].

In a related analysis of endogenous CO, it was noted that in septic human patients HO-1 expression as well as CO production was elevated in comparison to nonseptic critically ill patients [55]. More interestingly, it appears that survivors of sepsis had higher endogenous CO levels versus nonsurvivors [55]. Though we are lacking the definitive experiments which combine true polymicrobial sepsis and gaseous CO administration, the above mentioned work does suggest that CO may be therapeutically successful. We still lack data in septic or ALI/ARDS patients as to the safety or efficacy of CO in these critically ill patients. Prior to human studies, additional preclinical studies on large and small animal models are warranted, as discussed below.

## 4. Hyperoxia and Ventilator-Induced Lung Injury Models

ALI and ARDS are often a result of the inflammatory cascade triggered by intrinsic disease or an iatrogenic syndrome caused by the oxygen and mechanical ventilation physicians deliver to support tissue oxygenation. Experimental studies exploring the utility of CO administration during mechanical ventilation or during hyperoxygenation may become realistic if safe modes of delivery and therapeutic doses were established.

The lung damage resulting from hyperoxia exposure occurs predominantly in the respiratory endothelium (vessel lining) and epithelium (airway lining) [56]. Rats and mice exposed to hyperoxia (>95% oxygen) develop lung inflammation characterized by neutrophil influx, pulmonary edema, pleural effusion, and increased lung cell apoptotic markers. HO-1 is known to be increased in such models [56]. As a mimic of HO-1 overexpression, the concurrent delivery of CO at a concentration of 250 ppm in the hyperoxic environment prolongs survival of rats and mice subjected to a lethal dose of hyperoxia. CO administration also reduces histologic markers of lung injury such as neutrophil infiltration, fibrin deposition, alveolar proteinosis, pulmonary edema, and total apoptotic index. Mice subjected to this injury model were noted to have decreased expression of proinflammatory cytokines including TNF-*α*, IL-1*β*, and IL-6 [57]. Furthermore, hyperoxia and the resultant HO-1 increase are associated with activated MAPK in lung tissue. The protection by CO was dependent in this case on p38*β* MAPK and its upstream regulator mitogen activated protein kinase kinase 3 (MKK3) [45, 57]. It was also demonstrated that endothelial signal transducer and activator of transcription (STAT3) is essential for the protective effects of CO and HO-1 in oxidant induced lung injury and apoptosis [58]. Finally, it was shown in mouse lung endothelial cells exposed to hyperoxia that a low dose of CO at 250 ppm inhibited the initiation and propagation of extrinsic apoptosis pathways. The protective effect of CO in this model was dependent on ERK1/ERK2 MAPK [59].

Mechanical ventilation, even in the case of normoxia (21% oxygen), can induce ventilator-induced lung injury (VILI). Rat models of VILI utilizing co-administration of LPS and carbon monoxide demonstrated that CO was protective in this combined model of sepsis and lung injury. The protective effect was via a p38 MAPK pathway and independent of activator protein-1 and NF-*κ*B pathways [60]. A murine model of VILI which coadministered 250 ppm CO evaluated parameters of injury such as bronchial alveolar lavage (BAL) total protein, total cell count, and neutrophil count as well as induction of HO-1 and heat shock protein −70 in lung tissue. Though VILI generally results in increases in these parameters, the use of CO resulted in a decrease towards normal levels of BAL total protein, cell count and neutrophil count. CO also abolished early expression of the proinflammatory early growth response-1 protein and the cytoprotection by CO was dependent on the peroxisome proliferator-activated receptor protein-*γ*, an anti-inflammatory nuclear regulator [61]. Finally, consistent with the findings in smooth muscle relating the effects of CO to caveolin-1 and p38 MAPK [38], Hoetzel et al. demonstrated that CO protection in VILI is also dependent on caveolin-1 [39]. In contrast to the initiation of sepsis, which occurs at varying time points prior to clinical intervention, the fact that we control the initiation of mechanical ventilation and hyperoxia creates a unique window of therapeutic opportunity to concurrently initiate CO administration and perhaps more readily translate these experimental findings into benefits for critically ill patients.

## 5. Ischemia-Reperfusion Models

Cytotoxic ROS produced during ischemia-reperfusion (I-R) insult to the lung promote the recruitment of inflammatory leukocytes and cause lung injury and cell death secondary to both necrosis and apoptosis. Lung I-R injury (or anoxia-reoxygenation (A-R) injury in cells) is a model of transplant-induced tissue injury. Endothelial cells represent the primary target for ROS dependent injury. In a series of supporting experiments, HO-1^−/−^ mice were shown to be highly susceptible to lung I-R injury; as we might expect, CO can overcome this genetic susceptibility [62, 63]. A generalized understanding of the interaction between CO and I-R injury models is available from a wide variety of studies in other organ systems such as gut [64, 65], liver [66, 67], kidney [68–70], and heart [71, 72].

Our laboratory has used both mouse lung I-R injury as well as endothelial cell A-R injury to demonstrate that CO has potent anti-inflammatory and anti-apoptotic effects [26, 27]. In complementary studies of A-R in primary rat pulmonary artery endothelial cells and I-R injury to mouse lung, we noted the induction of caspase 3-dependent apoptosis by A-R or I-R. Treatment with CO diminished apoptosis in both models via a p38 MAPK-dependent pathway [27]. Expansion of this work demonstrated that CO activates the p38*α* MAPK isoform, and its upstream MAPK kinase MKK3, which modulates Fas-mediated cell death [26].

In a larger animal I-R model, miniature swine underwent left pulmonary artery and vein clamping for 90 minutes with or without 250 ppm CO supply during the procedure. The CO exposed group showed increased arterial oxygen tension, fewer pathologic radiographic findings, and decreased histologic markers of pathologic change at autopsy. BAL analysis revealed decreased inflammatory cell infiltrates (including neutrophils) and serum analysis demonstrated decreased inflammatory markers of IL-1*β* and IL-6 and high mobility box group-1 [73].

In a transplant model of I-R, CO (500 ppm) delivered at the end of a syngeneic orthotopic lung transplant in rats until a follow-up time of up to 6 days demonstrated CO mediated cytoprotection via anti-inflammatory action (downregulation of IL-6, MIP-1*α* and macrophage migration inhibitory factor). Anti-apoptotic effects in lung macrophages, endothelial, and epithelial cells were also observed [27]. Rat lung syngeneic transplant protection via 250 ppm CO delivered at timed intervals before and continuously after lung surgery demonstrated better graft function, as measured by partial pressure of oxygen, reduced neutrophil infiltration, maintenance of cellular ultrastructure, and reduced IL-6, IL-1*β*, TNF-*α*, inducible nitric oxide synthase, cyclooxygenase-2, and intracellular adhesion molecule-1. The CO effect in this model was NF-*κ*B independent and changes in IL-10 or HO-1 levels were not observed [74].

## 6. Complex and Large Animal Models

Several investigators have expanded the aforementioned work by creating large animal models and more complex clinical models. Dolinay et al. created a rat model in which combined insults of endotoxemia (via LPS) and VILI were administered in a stepwise lung injury model designed to mimic the common scenario of the intubated septic patient. Analysis of rat lungs, which had sequentially been exposed to 3 mg/kg intravenous LPS and then mechanical ventilation with tidal volumes of 12 mL/kg, revealed increased total protein, TNF-*α*, macrophages, and neutrophils in BAL fluid. Treatment with 100 and 250 ppm of CO concomitant to the ventilator injury revealed reductions in many of the above parameters as well as increases in the anti-inflammatory cytokine IL-10 and p38 MAPK activation [60].

Similar to systemic LPS, hind limb I-R with resultant small intestine inflammation has been used as a model of secondary organ damage in response to distant tissue injury. In this system, 250 ppm of CO administered at the time of limb reperfusion decreased ICAM-1 and TNF-*α* expression and diminished ICAM-1 dependent leukocyte adhesion as compared to the levels normally seen following I-R injury. Because it leads to severe intestinal injury and further inflammatory response, ICAM-1 dependent leukocyte venule adhesion in the small bowel is thought to be a gateway pathway to systemic inflammatory responses and multiorgan dysfunction [75].

Cynomolgus macaques exposed to LPS inhalation and either 250 or 500 ppm CO for 6 hours had some indications of reduction in airway inflammation. These reductions paralleled results seen with inhaled corticosteroid treatment (budesonide) as an established standard for airway anti-inflammatory treatment. Pulmonary neutrophilia was decreased by 500 ppm of CO. BAL fluid TNF-*α* was decreased by the 500 ppm of CO while IL-6 and IL-8 levels were unchanged. In comparison, 250 ppm CO had no effect on TNF-*α*, IL-6, or IL-8 [76].

Several studies in porcine models have shown the benefit of inhaled CO in treating endotoxemic shock induced by intravenous administration of LPS. Mazzola et al. administered LPS 40 *μ*g/kg/hour to large white pigs (swine) for four hours with and without one hour of CO pretreatment (250 ppm). Their combined studies demonstrated the ability of CO to improve lung mechanics as measured by airway resistance and parenchymal compliance; pulmonary edema was also decreased by CO inhalation. Furthermore, extrapulmonary organ effects of LPS were mitigated including preservation of heart stroke volume, kidney function, and liver function. Parameters of disseminated intravascular coagulation were decreased by CO pretreatment. Finally, CO decreased levels of proinflammatory IL-1*β* and increased levels of anti-inflammatory IL-10 [77, 78]. The major limitation of this work is the use of CO as a pretreatment which brings into question the clinical relevance for critical care as our opportunity to intervene is virtually always following the onset of shock. This limitation is addressed by Koulouras et al. in which pigs received inhaled CO starting 2.5 hours after the initiation of an intravenous LPS administration (20 *μ*g/kg/hour for 2.5 hours then 10 *μ*g/kg/hour for 3.5 hours). In these studies, a broad variety of cardiovascular parameters were monitored; LPS-induced increase in pulmonary artery pressure was the only parameter significantly altered by CO administration though there was a trend towards improvement in pulmonary vascular resistance. There were no changes in inflammatory markers with and without CO administration but there were however significant changes in alveolar cellular infiltration, edema, and hemorrhage [79]. Thus, studies using large and small animals demonstrate a trend towards improvement with CO administration.

## 7. Human Studies

As human studies are initiated, there are significant safety concerns given the somewhat narrow window of carbon monoxide dosing that would be necessary to avoid CO toxicity. Interestingly, safety trials demonstrate that CO inhalation up to 100 ppm for two hours, 500 ppm for one hour, or even 400 to 1000 ppm for approximately one hour is without adverse event and does not elevate carboxyhemoglobin (COHb) levels above those seen in heavy smokers [4–6]. One of the highest levels of CO delivered in a phase I trial setting is that of a recent single-blind, randomized, placebo-controlled phase 1 human trial evaluating drug safety and delivery. This trial utilized an investigational device the Covox DS (Ikaria, Clinton, NJ), which delivered 3.0 mg per kg CO per hour, for 1 hour given either once or daily for 10 days. COHb elevated reliably to 12% and no adverse effects were reported [7]. New CO delivery systems such as this one lend feasibility to future therapeutic trials with CO. CO delivery to critically ill patients on mechanical ventilation poses special challenges as well, given the potential devastating consequences of CO leaks to patients and staff.

Brief dosing of CO at 500 ppm for one hour after LPS administration to healthy human volunteers did not abrogate LPS-induced inflammatory responses, as indicated by no differences in plasma C-reactive protein, neutrophil count, TNF-*α*, IL-6, or IL-10. It seems likely that differences in CO binding, diffusion, and ultimately tissue delivery may account for the discrepancy between compelling mouse data using 250 ppm of CO and negative human data with 500 ppm of CO [4]. Notably parameters such as COHb vary between this study in humans (maximal COHb of 7.0%) [4] and a similar study in pigs (maximal COHb of 14.1%) [77]. However, studies using COHb as indicator of CO levels are limited by the fact that COHb does not reflect tissue CO levels and endogenous HO/CO activity nor is it predictive of CO toxicity [80].

Asthma is characterized by chronic airway inflammation; there has been well-justified interest in exploring the possibility that CO may have a therapeutic role for asthma patients. Murine asthma models using ova-albumin sensitization have improved BAL inflammatory cell counts (eosinophils and macrophages) when treated with CO. Exogenous CO administration reduced IL-5, proinflammatory mediators interferon-*γ*, leukotriene B4, and prostaglandin E2 [81]. Follow-up studies with a single 10-minute exposure to CO at 500 to 1000 ppm reduced methacholine-induced airway resistance in ova-albumin challenged mice as did repeated administration of low-dose CO 250–500 ppm over a 5-day period [82]. Observational human studies have suggested elevated levels of exhaled CO in nonsmoking asthmatics as compared to healthy control subjects [83] and there has been some suggestion that exhaled CO may have a role in characterizing asthma disease severity.

Patients with COPD are postulated to have disease progression related to a relative lack in HO-1/CO production relative to their non-COPD, smoking counterparts [84, 85]. Therefore, in a phase II clinical trial, Bathoorn et al. explored the efficacy of CO administration in abrogating the chronic airway inflammation that characterizes COPD. Administration of between 100 and 125 ppm for 2 hours on 4 consecutive days was safe with a maximal COHb level of 4.5% and led to a trend in reduction of airway responsiveness, as characterized by methacholine challenge, and a trend towards less sputum eosinophils [84]. In a similar example of what is believed to be a disease of chronic inflammation, a phase II trial of 100 to 200 ppm inhaled CO for two hours twice weekly for twelve weeks is being initiated in idiopathic pulmonary fibrosis patients; outcomes will include serum biomarkers of idiopathic pulmonary fibrosis, functional lung studies, and symptom questionnaires (NCT01214187).

## 8. Discussion

There may be therapeutic potential to CO-based interventions. The pleiotropic effects of CO in abrogating inflammation and apoptosis, and thus protecting against an array of cellular insults, suggest a great opportunity to intervene in multiple critical care illnesses—sepsis, ischemia, and ALI. We have extensive *in vitro* and *in vivo *evidence of the protective effects of CO. However, the field has not yet defined the precise pathways of CO action, raising a lurking concern that this therapy could have unexpected effects. The historical categorization of CO gas as a poison and the knowledge that it can be harmful at high doses bring this concern further to the fore. Despite success at safe doses of 250 to 500 ppm in rodent models, success in higher mammals and humans has been limited.

The amount of CO inhaled and/or exposure time are the most critical factors that determine the severity of CO toxicity. Administering inhaled CO poses challenges in controlling the absorption, distribution, and tissue targeting of CO. The characterization and implementation of CO carriers (CO-RMs) that deliver CO in a more controlled fashion may open opportunities for the design of CO-based pharmaceuticals in the future [86]. In addition, children and older adults are more susceptible to CO toxicity and may have more severe symptoms [87]. Predisposing conditions for CO toxicity have also been described, such as cardiovascular disease, COPDs or anemia [88]. Conceivably, ALI/ARDS patients, who already exhibit hypoxia and poor tissue oxygenation, would be even more susceptible to CO toxicity, even at low doses. Therefore, careful tailoring to different patient populations would be necessary if CO were to be used as a therapy. Dose ranges and kinetics have not been adequately assessed but should be areas of active inquiry. It may be that we have to return to phase I safety trials at higher CO doses or advance our delivery methods to attain higher CO concentrations in a given target organ.

Furthermore, we must consider the double-edged sword of a therapeutic entity that has such broad, potentially toxic, effects. There are valid concerns that blunting an inflammatory response, while beneficial in a sterile laboratory model of sepsis such as LPS administration, may be harmful in true infection and/or polymicrobial sepsis. These concerns are somewhat allayed by CLP- and CO-RM utilizing models which demonstrate not only benefit vis á vis blunted inflammatory response but also the promotion of bactericidal activity directly by CO, but there is more work to be done in defining this aspect of CO.

Another concern is the timing of CO administration, which in the well-developed transplant literature occurs before insult. This timing is not practical in critically ill patients because we cannot anticipate onset of severe illness. However, there is a growing body of research showing that co-administration and post-insult CO administration are effective. This is notable in the VILI literature in which CO can be administered during mechanical ventilation, which is the most likely scenario in critical care medicine. Notably, delayed CO administration, but not pretreatment, was beneficial in a mouse model of VILI [89]. Furthermore, the transplant literature has extended its studies and demonstrated benefit even in the setting of significantly delayed CO administration [90].

Nonetheless, CO administration is a novel and intriguing modality with great potential, if applied judiciously. CO therapy for critically ill patients suffering from lung injury, multiorgan failure, or organ transplantation rejection may yield results not previously attained in this exceptionally ill population. Compelling basic science investigations justify expanding CO to the clinical realm in the form of clinical trials. However, the specific cellular response to CO requires further elucidation and testing of CO in disease-specific animal models.

## Abbreviations

ALI: Acute lung injury
A-R: Anoxia-reoxygenation
ARDS: Acute respiratory distress syndrome
BAL: Bronchial alveolar lavage
Bcl-2: B-cell lymphoma-2
CLP: Cecal ligation and puncture
CO: Carbon monoxide
COHb: Carboxyhemoglobin
COPD: Chronic obstructive pulmonary disease
CO-RM: Carbon monoxide releasing molecule
ERK: Extracellular signal-regulated kinase
HO-1: Heme oxygenase-1
IL: Interleukin
I-R: Ischemia-reperfusion
JNK: c-Jun NH_2_-terminal protein kinase
LPS: Lipopolysaccharide
MAPK: Mitogen-activated protein kinase
MIP: Macrophage inflammatory protein
MKK3: Mitogen activated protein kinase kinase 3
NF-*κ*B: Nuclear factor-kappa-light-chain enhancer of activated B cells
PPM: Parts per million
PI3K/Akt: Phosphatidylinositol-3-kinase
ROS: Reactive oxygen species
STAT3: Signal transducer and activator of transcription 3
TNF-*α*: Tumor necrosis factor
VILI: Ventilator induced lung injury.
